# A Nomogram for Predicting Distant Metastasis Using Nodal-Related Features Among Patients With Nasopharyngeal Carcinoma

**DOI:** 10.3389/fonc.2020.00616

**Published:** 2020-05-29

**Authors:** Chuanbo Xie, Haojiang Li, Yue Yan, Shaobo Liang, Yanhong Li, Lizhi Liu, Chunyan Cui, Yuying Liu

**Affiliations:** ^1^Cancer Prevention Center, State Key Laboratory of Oncology in South China, Guangdong Key Laboratory of Nasopharyngeal Carcinoma Diagnosis and Therapy, Collaborative Innovation Center for Cancer Medicine, Sun Yat-sen University Cancer Center, Guangzhou, China; ^2^Department of Medical Imaging, State Key Laboratory of Oncology in South China, Guangdong Key Laboratory of Nasopharyngeal Carcinoma Diagnosis and Therapy, Collaborative Innovation Center for Cancer Medicine, Sun Yat-sen University Cancer Center, Guangzhou, China; ^3^Department of Radiation Oncology, Cancer Center, First People's Hospital of Foshan Affiliated to Sun Yat-sen University, Foshan, China

**Keywords:** nasopharyngeal carcinoma, distant metastasis, nomogram, nodal features, magnetic resonance imaging

## Abstract

Distant metastasis is among the main reasons for treatment failure in nasopharyngeal carcinoma (NPC) patients. To identify patients with a high risk of distant metastasis is important to guide posttreatment surveillance, appropriate time treatments, and prolonging their long-term survival. In this study, we systematically examined the associations between a series of nodal-related characteristics and distant metastasis-free survival (DMFS) by detailed MRI reading and established a nomogram for DMFS in NPC patients. T-stage, age group, Epstein–Barr virus (EBV) level, central nodal necrosis, and nodal number were identified as independent risk factors for distant metastasis and were included into the final nomogram. The calibration plot showed a high agreement between the prediction by the nomogram and actual observations. Our established nomogram achieved a high C-index in predicting distant metastasis in both of the training cohort (0.737) and the validation cohort (0.718). This nomogram incorporated several readily available nodal features from the MR images, and it might be useful for guiding clinical decision and NPC patients' posttreatment surveillance. It also provides cues for how to redefine N-stage. Additional research is needed to confirm our conclusions.

## Introduction

Nasopharyngeal carcinoma (NPC) is one of the most commonly diagnosed head and neck cancers in Southeast Asia, with a reported annual incidence of 30–80 cases per 100,000 people/years in endemic areas (1, 2). Distant metastasis is among the main reasons for treatment failure among NPC patients (3, 4). Therefore, identifying those at high risk of distant metastasis can help in prolonging their survival by formulating personalized posttreatment surveillance for timely interventions (5, 6).

Few studies have systematically examined the association between the prognostic values of magnetic resonance imaging (MRI)-based nodal features, such as nodal number, nodal grouping (NG), central nodal necrosis (CNN), and extracapsular spread (ECS), to NPC distant metastasis (7, 8). NG, defined as the presence of contiguous and symphysic lymph nodes (LNs), has been shown to be a direct indicator of regional nodal burden (9), suggesting that the tumor has broken the nodal network barrier and spread to distant organs (10, 11). CNN has been a biomarker for tumor hypoxia and radiotherapy resistance. It is often used to distinguish between benign and malignant LNs and for predicting metastasis risk (8). ECS is a histologic biomarker representing an aggressive biological nature of tumor cells and was found to be associated with increased risk of distant metastasis and shortening the overall survival (OS) of patients with head and neck cancers (12). However, its prognostic value in nasopharyngeal carcinoma has not yet been established. Retropharyngeal LNs (RLNs) are recognized as the “first echelon” nodes for NPC patients, and accumulating evidence has suggested that RLN metastasis may be an important prognostic factor for NPC patients' survival (13). As such, we hypothesized that grouping these above-mentioned factors together for estimating the risk of NPC distant metastasis might be a promising strategy to improve the accuracy of such prediction.

In this study, we aimed to examine the prognostic significance of MRI-based nodal features to the DMFS of NPC patients and to establish a nomogram that could improve the accuracy for predicting the risk of distant metastasis.

## Methods and Materials

### Patients

#### Primary Cohort

Consecutive patients with histologically proven NPC, treated at the Sun Yat-sen University Cancer Center (SYSUCC, Guangzhou, China) between January 2010 and 2013, were selected as the training cohort. On the basis of the patients' records, we selected those who had a complete pretreatment evaluation comprising a complete history, physical examination, hematology and biochemical profiles, nasopharyngeal and cervical MRI, chest X-ray, and abdominal ultrasonography. Additional inclusion criteria included those who (a) absent distant metastasis at the time of diagnosis; (b) underwent intensity-modulated radiation therapy (IMRT); (c) had MR images of the nasopharynx and cervical regions; and (d) had known pretreatment Epstein–Barr virus (EBV) level. Patients were excluded if they had (a) other malignant tumors; (b) failed to complete radiotherapy for physical reasons during the treatment; or (c) received targeted therapy, as this is not a standard treatment for NPC patients.

#### Validation Cohort

A total of 424 NPC patients who were treated at the First People's Hospital of Foshan Affiliated to Sun Yat-sen University (Foshan, China) from April 2010 to March 2014 were classified as the external validation cohort. The inclusion and exclusion criteria were the same as those for the training cohort.

The Institutional Review Board of both institutions approved this study. The authenticity of the study was validated by uploading the key raw data onto the Research Data Deposit (RDD) public platform (www.researchdata.org.cn), with the approval RDD number RDDA2018000928.

## Image Assessment and Criteria for Lymph Nodal Grouping

All patients underwent MRI examinations with a 1.5-Telsa superconducting system (CVi-EXCITE-II, American GE Company, Boston, United States) or a 3.0-Telsa superconducting system (Siemens Magnetom Tim Trio, Erlangen, Germany). T1–and T2–weighted MRI scans were performed axially, coronally, and sagittally in the conventional nasopharyngeal and cervical regions. The MR images were independently reviewed by two radiologists with over 10 years of experience in reading MR images for head and neck tumors. Any disagreements were solved by mutual discussion.

Clinical staging was performed according to the 8th edition of the American Joint Committee on Cancer (AJCC) staging system. The radiologists recorded the minimal diameter (MID) of the largest retropharyngeal and cervical LN and defined RLN metastasis as MID ≥ 5 mm and cervical LNs metastasis as MID ≥ 10 mm (or ≥11 mm for level II).

For cervical LNs' involvement, we further divided them into upper or lower levels. The former and latter were defined as metastatic LNs at levels I, II, III, and Va, and level IV and Vb, respectively, to the caudal border of the cricoid cartilage. NG was defined as the presence of three or more contiguous and symphysic LNs, each of them with a MID between 8 and 10 mm. CNN was defined as the occurrence of a centrally focal area of high signal on T2WI and of low signal on T1WI and enhanced T1WI sequence with or without peripheral ring enhancement. ECS manifested as the occurrence of LNs with indistinct margins, irregular capsular enhancement, or infiltration into adjacent tissue. The numbers of LNs for each patient were counted from the upper region to the lower region of the neck by two radiologists. When two or more nodes coalesced but could still be distinguished from each other, the diameter of each node was measured and counted as separate nodes. If the matted LNs could not be distinguished from each other, the diameter of the matted node was recorded and was counted as one LN.

## Measurement of Epstein–Barr Virus DNA

Pretreatment plasma EBV DNA (pre-DNA) levels were measured by the same real-time polymerase chain reaction in the central labs of the two hospitals. This method quantified the EBV level toward the *Bam*HI-W region of the EBV genome. The detailed methodology of plasma EBV DNA detected was as previously described (14) and was categorized into a low (<4,000 copies/ml) or high (≥4,000 copies/ml) EBV level groups for analysis on the basis of previously validated cutoff values (15).

## Therapy

Both cohorts of patients had whole-course IMRT, as previously described (16). Most of the patients also received concomitant or induction chemotherapy. Concomitant chemotherapy consisted of weekly cisplatin (30–40 mg/m^2^) regimen or two to three cycles cisplatin (80–100 mg/m^2^) within 21 days. Induction chemotherapy comprised two to three cycles of cisplatin (80 mg/m^2^) plus 5-fluorouracil (1,000 mg/m^2^); cisplatin (75 mg/m^2^) plus docetaxel (75 mg/m^2^); and cisplatin (60 mg/m^2^) and 5-fluorouracil (600 mg/m^2^) plus docetaxel (60 mg/m^2^) within 21 days.

## Follow-Up and Outcomes

The follow-up period started from the first day after radiotherapy to death or the last clinic visit. Patients were followed up every 3 months in the first to second year, then every 6 months in the third to fifth year, and once a year thereafter. The major outcome of this study was distant metastasis-free survival (DMFS), which was estimated from the day after radiotherapy completion to the day of last visit or distant failure occurred.

## Statistical Analyses

Percentages were used to describe categorical variables. The differences in the distribution of all factors between the primary and validation cohort were analyzed using the chi-square test. Kaplan–Meier analyses were performed to screen for potential clinical or nodal-related variables that could be used to establish the nomogram for predicting distant metastasis (i.e., *P* < 0.05). These variables were age group (<45 vs. ≥45 years), pre-DNA level (<4,000 vs. ≥4,000 copies/ml), T-stage (T1, T2, T3, and T4), chemotherapy (yes vs. no), laterality of RLN metastasis (no/unilateral vs. bilateral), upper cervical of LN metastasis (no/unilateral vs. bilateral), nodal metastasis of the neck region (no, upper region, and lower region), NG (yes vs. no), CNN (yes vs. no), ECS (yes vs. no), and the nodal numbers (in continuous). Age was categorized into <45-year-old group and ≥45-year-old group, as previous paper suggested that patients aged ≥45 years had poor survival (17). Then, we used stepwise method to select the above-screened variables that could be incorporated into the nomogram. The significance levels of the stepwise method for entry (SLE) and for stay (SLS) were 0.25 and 0.15, respectively. We evaluated the performance of the established nomogram for predicting distant metastasis using the Harrell concordance index (C-index) and calibration curve. A larger C-index indicated a more accurate prognostic value. All C-indexes and their 95% CI and *P*-values were generated by bootstraps with 1,000 resamples. The nomogram was validated using the external validation cohort. All analyses were conducted with the R software (version 3.0.2). A *P* < 0.05 was considered significant. All the tests were two-sided.

## Results

### Patients Characteristics

A total of 733 NPC patients were enrolled in the training cohort and 424 into the validation cohort. Table 1 shows the patients' characteristics in the training and validation cohorts. The proportion of patients with age more than 45 years was significantly higher in the primary cohort (59.0%) than in the validation (49.8%) cohort. Patients in the validation cohort were more likely to be diagnosed with advanced T-stage disease (28.8 vs. 24.6%) and with bilateral RLN metastasis (31.8 vs. 26.3%) but were less likely to have NG (19.8 vs. 23.6%) than in the training cohort. We also found that the proportion of patients who had bilateral CNN was significantly higher in the validation cohort (20.8 vs. 5.6%) than in the training cohort. Other clinical characteristics between these two cohorts were comparable.

**Table 1:** Sociodemographic and clinical characteristics of the participants.

	**No. of patients (%)**		
**Variables**	**Training cohort *n* = 733 (100%)**	**Validation cohort *n* = 424 (100%)**	***P*-value**
Age groups			
<45	368 (50.2)	174 (41.0)	0.003
≥45	365 (49.8)	250 (59.0)	
Sex			
Male	534 (72.9)	320 (75.5)	0.329
Female	199 (27.2)	104 (24.5)	
WHO histologic types			
I	5 (0.7)	0 (0.0)	<0.010
II	39 (5.3)	0 (0.0)	
III	689 (94.0)	424 (100.0)	
EBV level copies/ml			
<4,000	421 (57.4)	395 (93.2)	<0.010
≥4,000	312 (42.6)	29 (6.8)	
T-stage^*^			
T1	184 (25.1)	117 (27.6)	0.042
T2	87 (11.9)	57 (13.4)	
T3	282 (38.5)	128 (30.2)	
T4	180 (24.6)	122 (28.8)	
N-stage^*^			
N0	174 (23.7)	73 (17.2)	0.022
N1	398 (54.3)	236 (55.7)	
N2	105 (14.3)	82 (19.3)	
N3	56 (7.6)	33 (7.8)	
AJCC stage^*^			
I	68 (9.3)	36 (8.5)	0.810
II	157 (21.4)	98 (23.1)	
III	285 (38.9)	156 (36.8)	
IV	223 (30.4)	134 (31.6)	
Induction chemotherapy			
No	379 (51.7)	197 (46.5)	0.086
Yes	354 (48.3)	227 (53.5)	
Chemotherapy			
No	99 (13.5)	69 (16.3)	0.198
Yes	634 (86.5)	355 (83.7)	
Laterality of RLN metastasis			
No/unilaterality	540 (73.7)	279 (68.2)	0.050
Bilaterality	193 (26.3)	130 (31.8)	
Cervical lymph nodes metastasis	598 (81.6)	324 (76.4)	0.035
No/unilaterality			
Bilaterality	135 (18.4)	100 (23.6)	
Regions			
No	174 (23.7)	73 (17.2)	0.033
Upper region	505 (68.9)	318 (75.0)	
Lower region	54 (7.4)	33 (7.8)	
Nodal size
<6 cm	729 (99.4)	410 (96.7)	<0.010
≥6 cm	4 (0.6)	14 (3.3)	
Nodal grouping			
No	560 (76.4)	340 (80.2)	0.135
Yes	173 (23.6)	84 (19.8)	
Central nodal necrosis			
No/unilaterality	692 (94.4)	336 (79.3)	<0.010
Bilaterality	41 (5.6)	88 (20.8)	
Extracapsular spread			
No	653 (89.1)	384 (90.6)	0.426
Yes	80 (10.9)	40 (9.4)	

*EBV, Epstein–Barr virus; AJCC, American Joint Committee on Cancer; RLN, retropharyngeal lymph node*.

* *8th version of the TNM staging system*.

### Outcome

**Figure 1:**
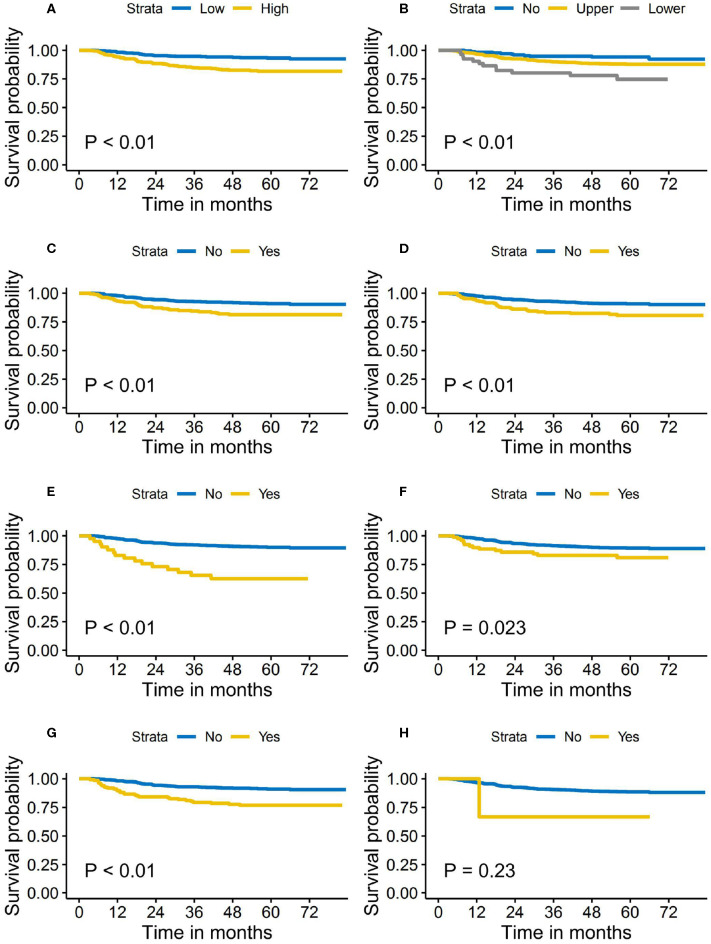
Distant metastasis-free survival stratified by the pretreatment Epstein–Barr virus (EBV) DNA level and nodal features. **(A)** Pretreatment EBV DNA level. **(B)** Cervical regions of lymph nodes metastasis. **(C)** Bilaterality of retropharyngeal lymph nodes' metastasis. **(D)** Nodal grouping. **(E)** Bilaterality of central nodal necrosis. **(F)** Extracapsular spread. **(G)** Bilaterality of cervical lymph nodes metastasis. **(H)** Nodal size >6 cm.

The median follow-up time for the primary cohort was 62.0 months (range, 1.4 to 83.4 months). A total of 82 cases developed distant metastasis, accounting for 11.2% of the entire study cohort. The 5-year DMFS was 88.0 and 85.6% for the patients in the training cohort and validation cohort, respectively. During the follow-up period, 78 (10.6%) patients in the training cohort and 83 (19.6%) in the validation cohort died. The loco-regional recurrence rates in the training and validation cohort were 9.8 and 8.3%, respectively. Figure 1 shows the Kaplan–Meier survival curves of DMFS stratified by pretreatment DNA level and each of the nodal features, respectively.

### Univariate and Multivariate Analyses

Table 2 shows the associations between the patients' characteristics and DMFS. Age group (<45 vs. ≥45 years), pretreatment DNA level (<4,000 vs. ≥4,000 copies/ml), T-stage, N-stage, CNN, and nodal number were independent risk factors for DMFS. Compared with patients with no metastasized LNs or unilateral CNN, patients with bilateral CNN had more than doubled risk of distant metastasis (HR = 2.1, 95% CI = 1.1–4.4). We also found that each nodal number increase was associated with a 1.1-fold risk of developing distant metastasis (HR = 1.1, 95% CI = 1.0–1.2). However, no significant association between nodal size (HR = 2.3, 95% CI = 0.3, 18.0) and nodal sites (HR = 1.3, 95% CI = 0.7–2.4) to the risk of distant metastasis was found after controlling for other nodal features such as nodal numbers, CNN, and NG.

**Table 2:** Association between clinical- and nodal-related characteristics and distant metastasis-free survival.

**Variables**	**Univariate cox regression analysis**		Multivariate cox regression analysis^*^	
	**HR (95% CI)**	***P*-value**	**HR (95% CI)**	***P*-value**
Age groups				
<45				
≥45	1.6 (1.1, 2.6)	0.028	1.6 (1.0, 2.5)	0.050
Sex				
Male				
Female	1.1 (0.7, 1.8)	0.582	1.2 (0.7, 1.9)	0.540
WHO histologic types				
I				
II	0.8 (0.1, 6.6)	0.828	0.6 (0.1, 5.2)	0.643
III	0.5 (0.1, 3.9)	0.548	0.3 (0.0, 2.3)	0.256
EBV level in copies/ml				
<4,000				
≥4,000	2.8 (1.8, 4.5)	0.001	1.8 (1.1, 3.0)	0.028
T-stage^*^				
T1				
T2	2.0 (0.8, 4.9)	0.138	1.4 (0.6, 3.5)	0.463
T3	2.0 (1.0, 4.1)	0.059	1.5 (0.7, 3.3)	0.264
T4	3.9 (1.9, 8.0)	0.000	2.6 (1.2, 5.6)	0.015
N-stage^*^				
N0				
N1	1.5 (0.8, 2.9)	0.257	1.2 (0.6, 2.4)	0.634
N2	4.0 (1.9, 8.1)	0.000	2.8 (1.3, 6.2)	0.012
N3	4.3 (1.9, 9.8)	0.001	2.9 (1.2, 7.0)	0.022
AJCC stage^*^				
I				
II	2.0 (0.4, 9.2)	0.376	1.3 (0.3, 6.3)	0.733
III	3.7 (0.9, 15.5)	0.073	2.4 (0.5, 10.5)	0.252
IV	7.6 (1.8, 31.3)	0.005	4.2 (1.0, 18.7)	0.057
Induction chemotherapy				
No				
Yes	1.5 (1.0, 2.3)	0.078	0.9 (0.6, 1.4)	0.638
Chemotherapy				
No				
Yes	3.3 (1.2, 9.0)	0.020	1.8 (0.6, 5.4)	0.270
Laterality of RLN metastasis				
No/unilaterality				
Bilaterality	2.2 (1.4, 3.4)	0.001	1.2 (0.7, 2.0)	0.528
Cervical lymph nodes metastasis				
No/unilaterality				
Bilaterality	2.9 (1.9, 4.6)	<0.0001	1.3 (0.7, 2.4)	0.433
Regions
No				
Upper region	2.0 (1.0, 3.7)	0.042	0.9 (0.4, 1.9)	0.837
Lower region	4.4 (2.0, 10.1)	0.000	0.7 (0.2, 2.4)	0.586
Nodal size				
<6 cm				
≥6 cm	3.2 (0.4, 22.7)	0.253	2.3 (0.3, 18.0)	0.432
Nodal grouping				
No				
Yes	2.2 (1.4, 3.4)	0.001	1.0 (0.5, 1.9)	0.899
Central nodal necrosis				
No/unilaterality				
Bilaterality	4.6 (2.7, 8.1)	<0.0001	2.1 (1.1, 4.4)	0.036
Extracapsular spread				
No				
Yes	1.9 (1.1, 3.4)	0.025	0.9 (0.5, 1.8)	0.850
Nodal number	1.1 (1.1, 1.1)	<0.0001	1.1 (1.0, 1.2)	0.088

*EBV, Epstein–Barr virus; AJCC, American Joint Committee on Cancer; RLN, retropharyngeal lymph node*.

* *8th version of the TNM staging system*.

### Development and External Validation of the Nomograms for Distant Metastasis

**Figure 2:**
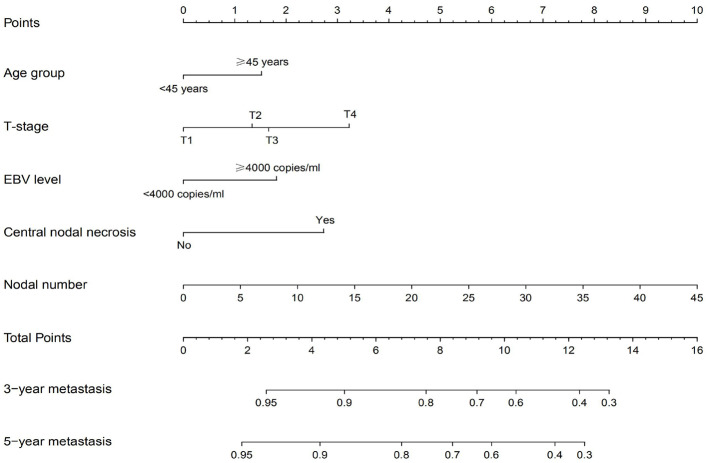
Nomogram for predicting distant metastasis using MRI-related nodal characteristics.

**Figure 3:**
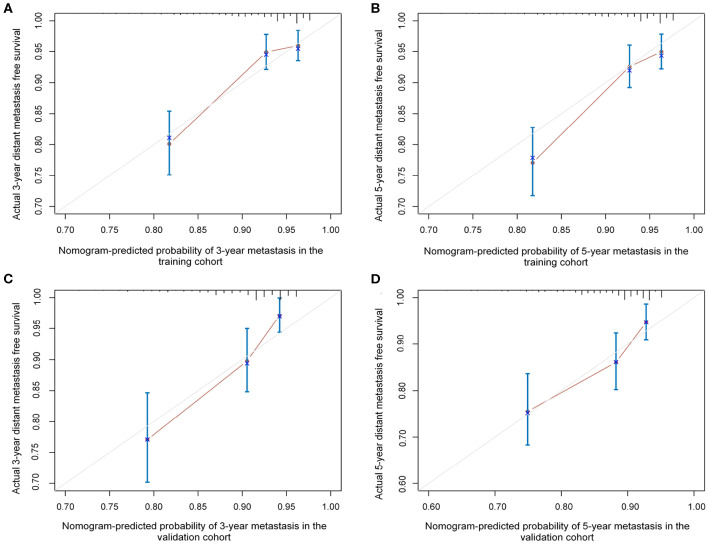
The calibration plots of the established nomogram in predicting the 3- and 5-year distant metastasis-free survival in the training cohort **(A,B)** and validation cohort **(C,D)**.

The independent prognostic factors for DMFS, namely, T-stage, age group, pretreatment DNA level, nodal number, and CNN, were used to construct the nomogram (Figure 2). The C-index of the nomogram in the training and validation cohort was found to be 0.737 and 0.718, respectively. Figure 3 shows the calibration plot of the nomogram for predicting the 3- and 5-year distant metastases of NPC. We found that our proposed nomogram had a high concordance between the predicted and observed 3- and 5-year distant metastases. In the validation cohort, the 3- and 5-year DMFS rates were 87.4 and 85.0%, respectively. The C-index of the nomogram for predicting DMFS was 0.718 in the external validation cohort.

## Discussion

In this study, we examined the prognostic significance of MRI-based nodal features to NPC patients' survival, based on which a nomogram for predicting the risk of distant metastasis was established. We found that nodal numbers and CNN were independent prognostic nodal features for predicting NPC patients' distant metastasis. The established nomogram demonstrated a high C-index in both the training and external validation cohorts, demonstrating its promising clinical significance.

Pretreatment EBV DNA level had been demonstrated as an important biomarker for clinical management of NPC patients (18). Peng et al. found that EBV DNA had an important prognostic value in predicting NPC patients' long-term survival after IMRT (19). Leung et al. also demonstrated that pretreatment DNA was an independent prognostic factor for OS and DMFS (15). Tang et al. incorporated pretreatment DNA into the nomogram for predicting DFS in NPC patients and found that pretreatment DNA could significantly improve the predictive value (14). The plasma EBV DNA originated from apoptotic and necrotic tumor cells; therefore, it is considered as a reliable biomarker for tumor burden. The optimal cutoff value for EBV DNA in predicting NPC patients' survival is still controversial. Previous studies had used 0, 1,500, 2,010, and 4,000 copies/ml to predict prognosis (15, 19, 20). In this study, we used 4,000 copies/ml to define EBV risk groups and confirmed that pretreatment DNA level was of important prognostic value in predicting NPC patients' DMFS. Our analysis supports to include pre-DNA level into the nomogram for predicting distant metastasis. However, in our supplementary analysis, we did not find that EBV level had a prognostic value in predicting OS after controlling for T-stage, RLN metastasis, and nodal number. We hypothesized that this could be partially explained by the high correlation between the nodal number and EBV level (*R* = 0.457, *P* < 0.001).

N-stage is commonly used to predict NPC patients' survival, especially DMFS. In the current 8th edition of the AJCC staging system, N-stage is defined according to nodal size and site. However, using these two variables to define N-stage might underestimate the cumulative effect of the nodal burden. This is because patients with only one metastatic LN are classified within the same N subgroup as those with 10, despite evidence suggesting that the latter is more likely to have distant metastases and regional relapse. Compared with size and sites, the number of positive LNs might be a better factor for representing nodal burden. To the best of our knowledge, our study is the first study to explore the prognostic value of LN number in NPC patients' survival. In line with other head and neck cancers, we found that the numbers of LNs was also of great clinical value for predicting NPC patients' survival.

CNN is shown as a focal area of high signal on T2-weighted MR image and as a region of low signal on T1-weighted MR images. The reported incidence of CNN among NPC patients ranges from 20 to 42%, and it is commonly used as an important imaging feature to distinguish between benign and malignant LNs (21–23). Consistent with the study by Lan et al. we found that CNN was an important independent prognostic factor for predicting the DMFS of NPC patients (7). Previous studies suggested CNN as a biomarker for tumor hypoxia, which had a negative impact on treatment effects. For instance, it was shown that hypoxic cells were less sensitive to radiotherapy and chemotherapy (24). In this study, we only found that bilateral but not unilateral CNN was associated with NPC patients' DMFS. This can be explained by the differed nodal burden between the two groups. We proposed that it might be necessary to include CNN into the model to predict DMFS in NPC patients.

Our study also explored the prognostic value of NG for distant metastasis and found that patients with NG had increased risk of distant metastasis than their counterparts without NG. NG may reflect a tendency that cancer cells had spread to multiple regions and distant LNs. There may be molecular and biological behavior differences between primary LNs and grouping nodes (25). However, after including other factors such as nodal number, pretreatment DNA level, and CNN into the model, the effect of NG was attenuated to null. This phenomenon can be attributed to the high correlation between NG and nodal numbers. If the radiologists found that counting nodal number was difficult, they can use NG as an alternative to predict long-term survival in NPC patients.

RLNs are the first echelon LN of metastasis in NPC patients. The importance of RLN in predicting NPC patients' survival has increasingly been recognized (26, 27). Tang et al. used the minimum diameter (>5 mm) as the criteria to define RLN metastasis and reported that it was significant for predicting distant metastasis regardless of its laterality (26). However, in this study, we found that patients with bilateral RLN metastasis had inferior survival than those without or with unilateral metastasis. This can be explained by the different characteristics of the participants between these two studies. But after including age group, nodal number, pre-DNA level, and nodal necrosis into the Cox regression model, RLN metastasis was not an independent prognostic factor for DMFS, which can be attributed to the collinearity between these variables. Similar to RLN metastasis, we found that patients with bilateral cervical LN metastasis had a significantly higher risk of DMFS than their counterparts without or with unilateral cervical LN metastasis. Like other squamous cell carcinomas of the head and neck, lymphatic drainage of the nasopharynx is predominant to cervical LNs. When cervical LNs had metastatic cancer cells, the primary cancer is very likely to have broken the nodal network of the neck and head regions and spread to distant organs.

A novel finding of this study was that nodal size and regions of neck metastasis were not included in the final nomogram. These two nodal variables were commonly used to define N-stage and predict posttreatment survival. Nodal size >6 cm was not included in the final nomogram because only a very small proportion (<0.1%) of patients had nodal size >6 cm, which might limit the power to detect its effect. This also suggests that the criteria of 6 cm for defining N3 stage and predicting distant metastasis should be reconsidered. That the region of nodal metastasis (upper and lower regions of the neck) was not included in the final nomogram can be attributed to the collinearity between it and the number of LNs. Our results also suggest that the current AJCC N-staging system should be redefined according to not only the nodal size and site characteristics but also other nodal features such as CNN and number of LNs.

The limitations of this study are described as follows. First, the sample size of this study was relatively small, and we could not rule out the possibility that our findings were chance only. Second, the investigated cases were all of Chinese Han origin. Therefore, cautions are warranted prior to generalizing these study findings to wider population.

In conclusion, we have established a concise nomogram comprising easily available MRI-based nodal features for predicting the risk of distant metastasis in NPC patients. It can be used for guiding clinicians in decision making and personalizing NPC patients' posttreatment surveillance. It also provides cues for how to redefine N-stage. Additional research is needed to further confirm our conclusions.

## Data Availability Statement

The datasets used or analyzed in the present study are available from the corresponding author upon reasonable request. The key raw data have been deposited into the RDD (http://www.researchdata.org.cn), with approval number RDDA2018000928.

## Ethics Statement

The studies involving human participants were reviewed and approved by The Institutional Review Board of Sun Yat-sen University Cancer Center. The patients/participants provided their written informed consent to participate in this study.

## Author Contributions

YYL and CC had full access to all the data in the study and take responsibility for the integrity of the data and the accuracy of the data analysis. Concept and design were done by all authors. Acquisition, analysis, or interpretation of data was carried out by CX, HL, SL, LL, CC, and YHL. Drafting of the manuscript was performed by CX. Critical revision of the manuscript for important intellectual content was carried out by CX, CC, and YYL. Statistical analysis was carried out by CX and HL. LL obtained the funding. Administrative, technical, or material support was provided by all authors. YYL and CC supervised the study.

## Conflict of Interest

The authors declare that the research was conducted in the absence of any commercial or financial relationships that could be construed as a potential conflict of interest.
